# 

**Published:** 1881-08

**Authors:** 


### INDEX.

### A.

PAGE.
A New Medical Journal, . . . . . 238
Aneurism, Aortic, by Chas. C. F. Gay, M. D., . . . . . . . . . . 552
Anus, Case of Imperforate, Reported by Herman Mynter, M. D., . . . . . 550
A Medical Bookstore, . . . . . . 238
Abbott, F. W., Dr., on Otorrhœa, . . . 97
Abbott, Frank W., M. D., on Some Practical Hints on the Fitting of Spectacles, 1
Acneiform Rash, Treatment of, . . 317
Acute Psoitis, by Herman Mynter, M. D., 202
Albuminuria, Diabetes Meditus and, . 373
Alkalinity of the Blood, . . . . . 31
Alumni Association of the Albany Medical College, . . . . . . . 45
Anæmla, Pernicious, . . . . . . 468
Andrews, Judson B., M. D., on Peritysphlitis, or Perinephritis, . . . . . 247
Angina Pectoris, by H. R. Hopkins, M. D., . . . . . . . . . . . 193
Anti-Vaccinationists, . . . . . . 324
Antipruritic Remedy, Note on a new, . 321
Antiseptic Osteotomy of the Tibia, . . 85

### B.

Ball in the Dead Body, The Difficulty in Finding a, . . . . . . . . . 176
Bartow, Bernard, M.D., on Litholaparxy, 168
Bartow, Bernard, M. D., on Elbow Fractures, . . . . . . . . . . 107
Blackham, Geo. E., M. D., F. R. M. S., on The Deliverances of the Retina, . 529
Blood, Alkalinity of the, . . . . . 31
Blood-Corpuscles, The formation of, . 466
Boardman, John, M. D., on Treatment of Herpes Zoster, . . . . . . . 257
Book Agents, A Word about, . . . 43
Brain, Foreign Substances Introduced into the, with Impunity, . . . . 38
PAGE.
Brewer, George E., on Case Illustrating the Advantages of Median Lithotomy, 440
Buffalo Medical College, . . . . 379
Buffalo College of Physicians and Surgeons, . . . . . . . . . . 89
Buffalo State Insane Asylum, . . . 41

### C.

Cary, Dr. Walter, . . . . . . 190
Cancerous Stomach, Successful Case of Resection of, . . . . . . . 37
Cerebral Tumor Involving the Nerves of the Special Senses, by Lucien Howe, M. D., . . . . . . . . . . 299
Cephalic Version in the Postural Position, by Dr. John Hauenstein, . . 289
Charbon, Pasteurs' Experiments on, . 372
Chlorate of Potash Poisoning, . . . 319
Chronic Prostatic Enlargement, Treatment of, . . . . . . . . . 230
Clinics at the Buffalo General Hospital, reported by C. C. Fredericks, M. D., . 253
Clinics at the Buffalo General Hospital, reported by C. C. Fredericks, M. D., . 212
Code of Ethics, . . . . . . . 377
Consultation, Three Views of a, . . 40
"Congestion of the Brain," Is it a Correct Pathological Expression? . . 367
Conjunctivitis, Nascent Iodide of Silver in the Treatment of, . . . . . . 318
Compound Dislocation, reported by Dr. C. C. F. Gay, . . . . . . . . 515
Consumption, Relation of Foul-Air to, . 32
Convulsions, Puerperal, reported by H. R. Hopkins, M. D., . . . . . . 11
Correspondence and Clinical Reports, . 137
Corrosive Sublimate in Dysentery, . 38
Council of the University of Buffalo, . 379
Cremation, . . . . . . . . . 79
Cremation in Denmark, . . . . 131

PAGE.

Croup, Treated by Passing Catheter into the Trachea by the Mouth, . . . 130

**D.**

Davidson, A. R., M. D., on "Sewer Gas" and Its Dangers, . . . . . . 537
Daniels, C. M., M. D., on Report on Last Ovariotomy, performed by Prof. J. P. White, . . . . . . . . . . 159
Daniels, C. M., M. D., on An Objection to "Listerism," . . . . . . 512
Decision of the Hon. George Barker, . 49
Diabetes Mellitus and Albuminuria, . 373
Diseases, The Relation of, by P. W. Van Peyma, . . . . . . . . . 60
Dysmenorrhœa and Sterility, Dilatation of the Cervical Canal for Spasmodic, . 403

**E.**

Elbow Fractures, reported by Bernard Bartow, M. D., . . . . . . . . 107
Ellis, Dr. L. E., on Twin Labor at Seven Months, . . . . . . . . . 219
Epilepsy, Treatment of, . . . . , 517
Erie County Medical Society, . . . 274
"Erysipelas" of the Face, Treatment of Frequently Recurring, . . . . . 38
Examination into the Department of the Interior, . . . . . . . . . 235
Eye, Remarks on Syphilitic Affections of the; Translated from the French by Lucien Howe, M. D., . . . . . 443

**F.**

Feet, Fœtid Sweating of the, . . . 322
Female Doctors, . . . . . . . . 77
Fœtid Sweating of the Feet, . . . 322
"Fooled by Temperature," . . . 82
Foreign Bodies in the Intestines, by Thomas Lothrop, M. D., . . . . . 346
Fothergill, J. Milner, M. D., on The Tongue, . . . . . . . . . 24
Foul-Air, Relation to Consumption, . 32
Fumigation, Local Mercurial, . . . 322
Fracture of the Lumbar Vertebræ, . . 112
Fredericks, C. C., M. D., on Surgical Cases, . . . . . . . . . . 111
Fredericks, Dr. C. C., Report of Clinics at the Buffalo General Hospital, . . 212
Fredericks, C. C., M. D., Report of Clinics at the Buffalo General Hospital, . . 253

PAGE.

**G.**

Gay, Chas. C. F., M. D., on Aortic Aneurism, . . . . . . . . . . 552
Gay, C. C. F., M. D., on Compound Dislocation, . . . . . . . . . 515
Gay, C. C. F., M. D., on Pelvic Version, 215
Goitre, Treatment of, with Ergotin, . 448
Gould, William, M. D., Obituary Notice, 429
Gouty Heart, A case of, reported by H. R. Hopkins, M. D., . . . . . . 255

**H.**

Heath, Dr. W. H., on Hernia, . . . 241
Hæmorrhage, Post-Partum. . . . 470
Hauenstein, Dr. John, on Cephalic Version in the Postural Position, . . 289
Hernia, Strangulated Umbilical, by Herman Mynter, M. D., . . . . . . 402
Hernia, The Permanent Cure of, by W. H. Heath, M. D., . . . . . . 241
Hopkins, Dr. H. R., Letters from, . . 133
Hopkins, H. R., M. D., on A Case of Gouty Heart, . . . . . . . 255
Howe, Lucien, M. D., Translation of Remarks on Syphilitic Affections of the Eye, . . . . . . . . . . 443
Howe, Lucien, M. D., on The Use of the "Radiometer" in Testing the Vision, . . . . . . . . . 210
Howe, Lucien, M. D., An Observation Concerning Catarrhal Inflammation of the Eustachian Tube, . . . . . 160
Howe, Dr. Lucien, M.D., report of a Case of Cerebral Tumor Involving the Nerves of the Special Senses, . . . 299
Huxley's Theory of Disease, . . . 234
Hygienic, . . . . . . . . . 1[illegible]9
Hypertrophy and Dilatation of the Heart during Scarlet-Fever Nephritis, . . 316

**I.**

Impotence, Psychical, . . . . . 29
Incubation in Various Diseases, . . 472
Infants, Artificial Nourishment of, . 36
International Medical and Sanitary Exhibition, . . . . . . . . . 88
International Medical Congress, . . 91
Intestinal Obstruction caused by Impacted Gall-stone, . . . . . . 226
Inunction of Sapo Viridis in Scrofula, . 315

PAGE.

Inversion of the Uterus, Report of a Case of, by James P. White, M. D., . 146
Iodoform as a Dressing for Wounds, . 312
Iodoform, Unpleasant Results from the Use of, . . . . . . . . . 461
Irrigation of the Uterus Immediately after Delivery. Translated by P. W. Van Peyma, M. D., . . . . . . 220

L.

Lactation, Effects of Drugs in, . . . . 172
Lichen Ruber, Cure of, by Hypodermic Injections of Arsenic, . . . . . 323
"Listerism," An Objection to, by C. M. Daniels, M. D., . . . . . . . . 512
Litholapaxy, by Bernard Bartow, M. D., 168
Lithotomy Median, Case Illustrating the Advantages of, by George E. Brewer, 449
Locomotor Ataxy, Syphilis and, . . 222
Lothrop, Thomas, M. D., on Obstruction of the Bowels, . . . . . . . . 302
Lothrop, Thomas, M. D., on Foreign Bodies in the Intestines, . . . . . 346

M.

Malaria, The Pathology of, . . . . 446
Malaria, The Pathology of, . . . . 362
Maltine, Dr. J. Milnor Fothergill on Use of, . . . . . . . . . . . . 238
Mann, Matthew D., A. M., M. D., on Rupture of the Perineum in Labor; Its Prevention and Treatment, . . 498
Mann, Matthew D., A. M., M. D., on Two Cases of Ovariotomy Successfully Performed, . . . . . . . . . 351
Malpractice in Belgium, An Action for, 129
Marcley, J. T., M. D., on Variola in Buffalo, . . . . . . . . . 337
Medical Profession, The American, . 427
Micrococcus of Syphilis, Discovery of the, . . . . . . . . . . 83
Milk, How It Should be Taken, . . 34
Mirth, Hygienic Value of, . . . . . 33
Mycosis of the Trachea, . . . . . 35
Mynter, Herman, M. D., on Urethrotomia Externa, . . . . . . . . . 67
Mynter, Herman, M. D., on Acute Psoitis, . . . . . . . . . . . 202
Mynter, Herman, M. D., Report of a Case of Imperforate Anus, . . . 550

PAGE.

Mynter, Herman, M. D., report of Surgical Cases, . . . . . . . . 163
Mynter, Herman, M. D., Translation on The Medico-Legal Import of the Opium Habit, . . . . . . . . 14
Mynter, Herman, M. D., on Strangulated Umbilical Hernia, . . . . . . 402

N.

Nascent Iodide of Silver in the Treatment of Conjunctivitis, . . . . 318

O.

Observation Concerning Catarrhal Inflammation of the Eustachian Tube, by Lucien Howe, M. D., . . . . . 160
Obstetrical Forceps, Extracts from a Paper on the, by Prof. James P. White, M. D., . . . . . . . . . . . 156
Obstruction of the Bowels, by Thomas Lothrop, M. D., . . . . . . . . 302
Oil of Tansy, Effects of, . . . . . 232
Opium Habit, The Medico-Legal Import of the; Translation by Herman Mynter, M. D., . . . . . . . . . 14
Organic Forms, The Artificial Production of, . . . . . . . . . . 471
Otorrhœa, by F. W. Abbott, M. D., . . 97
Ovariotomy and Parotitis, . . . . 36
Ovariotomy Performed by J. P. White, Report on last, by C. M. Daniels, M. D., 159
Ovariotomy Successfully Performed, Two Cases of, by M. D. Mann, A. M., M. D., . . . . . . . . . . . 351
Oxygen and Protoplasm, by W. H. Ritt, M. D., . . . . . . . . . . 396

P.

Parotitis, Ovariotomy and, . . . . 36
Pasteurs' Investigations on Rabies, . 228
Pelvic Version, by C. C. F. Gay, M. D., 215
Perinephritis, or Perityphlitis, by Judson B. Andrews, M. D., . . . . . 247
Perityphlitis or Perinephritis, by Judson B. Andrews, M. D., . . . . . . 247
Phagædemic Ulcerations, Pyrogallic Acid in, . . . . . . . . . 321
Pitt, W. H., M. D , on Oxygen and Protoplasm, . . . . . . . . . 396
Poisoning, by Chlorate of Potash, . . 319

PAGE.
Pregnancy; Coition in, . . . . . 19
Pryor, J. H., M. D., Report of a Case of Typhoid Fever with a Peculiar Complication, . . . . . . . . . 555
Puerperal Convulsions, . . . . . 175
Puerperal Hemorrhage, Treatment of, . 261
Pupil, Symptomatology of the, by Lucien Howe, M. D., . . . . . . . . 433
Pupil, Symptomatology of the, by Dr. Lucien Howe. Part II, . . . 481
Pyrogallic Acid in Phagædemic Ulceration, . . . . . . . . . . 321

Q.

Quack Advertisements, . . . . . 81

R.

Rabies, Pasteurs' Investigations on, . 228
"Radiometer" in Testing the Vision, on the Use of the, by Lucien Howe, M. D., 210
Rapid Breathing, . . . . . . . . 72
Refrigerators for Sick Chambers, . . 171
Regents, The Board of, and Medical Colleges, . . . . . . . . . . 375
Retina, Deliverances of the, . . . 385
Retina, The Deliverances of the, by Geo. E. Blackham, M. D., F. R. M. S., . . 529
Remarkable Case of Maternity, . . 81
Remarks on Pathology, by Samuel Wilkes, M. D., F. R. C. S., . . . 114
Resection of the Bowels, A Successful Case of, . . . . . . . . . 69
Rheumatism, Alterations of the Nerves in Chronic, . . . . . . . . 374
Risks of Intra-Pleural Injections, . . 519
Roehrig, Dr., on Self-Adjusting Shell-Plate Eutropium Forceps, . . . 401
Rupture of the Perineum in Labor; Its Prevention and Treatment, by Matthew D. Mann, A. M., M. D., . . . . 498

S.

Salicin and the Salicylates in Rheumatism, The Debate on, . . . . . 365
Scrofula, Inunction of Sapo Viridis, . 315
Separation of the Epiphysis of the Lower End of the Humerus, . . . . . 110
"Sewer Gas" and its Dangers, by A. R. Davidson, M. D., . . . . . . 537
Shell-Plate Entropium Forceps, Self-Adjusting, by Dr. Roehrig, . . . 401

PAGE.
Society of the Red Cross, . . . . . 378
Soothing Ointments and the Indication for their Use, . . . . . . . 321
Soil, Sanitary Relations of the, . . 450
Spectacles, Some Practical Hints on the Fitting of, by Frank W. Abbott, M. D., 1
Subcutaneous Nævi, A New Method of Treating, . . . . . . . . . 171
Successor of Prof. James P. White, . 276
Sulphide of Calcium for Suppurating Buboes, . . . . . . . . . 317
Surgical Cases, reported by C. C. Fredericks, M. D., . . . . . . . . . 111
Surgical Cases, by Herman Mynter, M. D., . . . . . . . . . . 163
Syphilis and Locomotor Ataxy, . . 222
Syphilis Hereditary, Translated by P. W. Van Peyma, M. D., . . . . . 258
Syphilis and Tabes, . . . . . . 370
Syphilitic Chancre, Effects of Excision of the, . . . . . . . . . . 132

T.

Tabes and Syphilis, . . . . . . 370
Temperature, Case of Remarkably low, 35
Tongue, by J. Milner Fothergill, M. D., 24
Trachea, Mycosis of the, . . . . . 35
Treatment of Syphilis by Subcutaneous Injections, . . . . . . . . . 356
Twin Labor at Seven Months, by Dr. L. E. Ellis, . . . . . . . . . 219
Typhoid Fever, The Treatment of, . . 404
Typhoid Fever, A Case of, with a Peculiar Complication, Reported by J. H. Pryor, M. D., . . . . . . . . 555

U.

Urethrotomia Externa, by Herman Mynter, M. D., . . . . . . . . . 67

V.

Vaccination a Relic of Barbarism, . . 323
Vaginal Chancres, . . . . . . 310
Van Peyma, P. W., M. D., Translation on Hereditary Syphilis, . . . . . 258
Van Peyma, P. W., M. D., on The Relation of Diseases, . . . . . . 60
Van Peyma, P. W., M. D., Translation on Irrigation of the Uterus Immediately after Delivery, . . . . . . . . 220

PAGE.

Variola in Buffalo, by J. T. Marcley, M. D., . . . . . . . . . . . 337

Vaso-Motors of the Lymphatics, . . . 374

Venesection, The Therapeutics of, . . . 519

Vomiting in Pregnancy, . . . . . 313

### W.

White, Prof. James P., M. D., Extracts from a Paper on the Obstetrical Forceps, . . . . . . . . . 156

White, James P., M. D., Report of a Case of Inversion of the Uterus, . . . . 145

PAGE.

White, Professor James P., Death of, . 137

White, Prof. James P., M. D., Obituary, 183

Whooping Cough, The Diagnosis and Treatment of, . . . . . . . . 121

Wilkes, Samuel, M. D., F. R. C. S., Remarks on Pathology, . . . . . 114

Will Guiteau Receive the Benefit of a Doubt? . . . . . . . . . 236

Wounds of the Intestines made During Life, The Characteristic Appearance of, . . . . . . . . . . . 371

Writer's Cramp, . . . . . . . . 374

